# Club cell secretory protein (CC16) polymorphisms in preterm neonates with respiratory distress syndrome and bronchopulmonary dysplasia

**DOI:** 10.1007/s00431-025-06169-7

**Published:** 2025-05-15

**Authors:** Dimitrios Rallis, Petros Bozidis, Marianthi Sotiropoulou, Georgia Ragia, Maria Baltogianni, Niki Dermitzaki, Eleni Maragoudaki, Vangelis G. Manolopoulos, Konstantina Gartzonika, Katerina Antoniou, Vasileios Giapros

**Affiliations:** 1 Neonatal Intensive Care Unit, School of Medicine, University of Ioannina, Stavrou Niarchou Avenue, Ioannina, Greece; 2 Microbiology Department, School of Health Science, Faculty of Medicine, University of Ioannina, Ioannina, Greece; 3 School of Pharmacology, University of Ioannina, Ioannina, Greece; 4 Laboratory of Pharmacology, Medical School, Democritus University of Thrace, Komotini, Greece; 5 Individualised Medicine & Pharmacological Research Solutions (IMPReS) Center, Alexandroupolis, Greece; 6 Clinical Pharmacology Unit, Academic General Hospital of Alexandroupolis, Alexandroupolis, Greece; 7 Present Address: Laboratory of Pharmacology, School of Pharmacy, Aristotle University of Thessaloniki, 54124 Thessaloniki, Greece

**Keywords:** Alveolar injury, Mechanical ventilation, Single-nucleotide polymorphisms, Pneumoprotein, Prematurity

## Abstract

To investigate the genotype frequencies of club cell secretory protein (CC16) polymorphisms in preterm neonates and explore their association with respiratory distress syndrome (RDS), and bronchopulmonary dysplasia (BPD). A prospective cohort study was conducted including 187 preterm neonates of ≤ 34 weeks of gestational age. Genotype frequencies of rs4963506, rs12270961, and rs3741240 CC16 polymorphisms were detected, comparing the CC16 polymorphisms between neonates with versus without RDS, and with versus without BPD. Serum CC16 was measured, when available, on the first and fourteenth postnatal days. A significant association of increased risk of RDS was detected for the homozygous GG rs4963506 variant (OR 3.03, 95%CI 1.36–6.75, *p* = 0.021), and the homozygous GG rs12270961 variant (OR 2.88, 95%CI 1.31–6.28, *p* = 0.024), but not for the homozygous GG rs3741240 variant, after adjusting for gestational age, mode of delivery, and antenatal administration of steroids. The homozygous GG rs4963506 variant was also associated with higher serum levels of CC16 compared to the GA/AA rs4963506 variants, on the first postnatal day. No associations between BPD and the genotype frequencies of any polymorphisms were detected. *Conclusion*: In preterm neonates, the homozygous GG rs4963506 and rs12270961 variants were both substantially associated with a higher risk of RDS. Further studies are warranted to validate our findings and explore the potential role of CC16 polymorphism detection in neonates at risk of respiratory morbidities.

**Table:** 

**What is Known:** ∙ Serum club cell secretory protein (CC16) reflects the lung’s developmental maturation and healing mechanisms for tissue damage. ∙ The CC16 polymorphisms have been inconsistently associated with lung function in children. ∙ No evidence exists regarding the association between CC16 polymorphisms and respiratory morbidity in neonates.	
**What is new:** ∙ Homozygous GG rs4963506 and GG rs12270961variants were associated with increased risk of respiratory distress in neonates. ∙ Homozygous GG rs4963506 variant was also associated with higher serum levels of CC16 on the first postnatal day. ∙ No associations between BPD and the genotype frequencies of any polymorphisms were detected.

**Supplementary information:**

The online version contains supplementary material available at 10.1007/s00431-025-06169-7.

## Introduction

The club cell secretory protein (CC16) is encoded by the *SCGB1 A1* gene on chromosome 11q12.3 and is highly expressed in the lungs [1]. Non-ciliated club cells secrete CC16 in large quantities which appears to be beneficial, due to its anti-inflammatory, antioxidant, immunomodulatory, and airway-repairing properties [1–3]. The concentration of serum CC16 reflects the lung’s developmental maturation and healing mechanisms for tissue damage [4–6]. Alveolar injury in neonates with respiratory diseases may trigger a proinflammatory response leading to increased CC16 synthesis and release in alveoli [5], whereas bronchoalveolar-blood barrier leakage may raise serum CC16 concentrations [5, 7].

CC16 protein levels are influenced by CC16 polymorphism rs3741240 [8, 9], while several polymorphisms including rs4963506 and rs12270961 have been examined in adults with respiratory disease [10]. Polymorphisms rs4963506, rs12270961, and rs3741240, are located in positions chr11:62,420,194 (GRCh38.p14), chr11:62,419,797 (GRCh38.p14), and chr11:62,419,070 (GRCh38.p14), with minor allele frequencies of 0.144, 0.19, and 0.30, respectively. The most investigated CC16 polymorphism is rs3741240, which is characterized by an adenine to guanine substitution at position 38 (A38G) and has been associated with reduced transcriptional activity of the gene activity [11]. According to previous studies, children with AA and AG genotypes showed a significantly lower serum CC16 levels compared to GG genotype carriers [12, 13]. The rs3741240 risk allele AA has been inconsistently associated with lung function in children [14, 15]. Moreover, polymorphisms rs3741240, rs4963506, and rs12270961 were examined in adults suffering from asthma and it was found that rs12270961 was associated with uncontrolled asthma [10]. In neonates, a few studies have examined the kinetics and predictive role of CC16 protein in relation to respiratory distress syndrome (RDS) and bronchopulmonary dysplasia (BPD) [2, 3, 5–7, 16, 17]; however, no evidence exists regarding the association between CC16 polymorphisms and respiratory morbidity in this population.

Based on the above concept, the aim of the present study was to investigate the genotype frequencies of CC16 polymorphisms rs4963506, rs12270961, and rs3741240 in a cohort of preterm neonates and explored their association with RDS and BPD.

## Material and methods

### Participants, clinical assessment and measures

A prospective cohort study was conducted at the University Hospital of Ioannina, from December 2020 to July 2022 enrolling 187 preterm neonates of ≤ 34 weeks of gestational age. An analysis was performed between neonates with RDS compared to neonates without RDS, and neonates who developed BPD compared to neonates without BPD. The data were anonymously recorded, and the study was approved by the Ethical Committee of the Institution (No 981/21.12.2020).

Neonatal RDS was diagnosed based on clinical and radiological evidence and managed according to the latest European Consensus Guidelines [18]. Surfactant (Curosurf, Chiesi, Farmaceutisi SPA, Parma, Italy) was administered at a dose of 200 mg/kg when FiO_2_ ≥ 0.3 was required to maintain SpO_2_ 88–92%, or when intubation in the delivery room was performed for primary resuscitation [18]. BPD was defined according to standard criteria [19].

The perinatal and neonatal characteristics that were recorded included gestational age, birth weight, sex, mode of delivery, small for gestational age (defined by a birth weight below the 10 th centile according to Fenton growth charts), maternal clinical chorioamnionitis (defined by the ACOG criteria), pregnancy-induced hypertension, maternal gestational diabetes, multiparity, mode of conception, complete course of antenatal steroid administration, prolonged rupture of membranes, Apgar scores, RDS manifestation, the need for mechanical ventilation, surfactant administration, patent ductus arteriosus, late onset sepsis (according to European Medicines Agency criteria [20]), retinopathy of prematurity (according to standard criteria [21]), the development of BPD, and the length of stay.

### Genotyping of CC16 polymorphisms

Blood samples for CC16 polymorphism detection were obtained within the first twelve hours of age. Whole blood samples were stored in EDTA at −80 °C, until the completion of the collection and the subsequent analysis.

DNA was extracted from 200 μL of whole blood using the MagMAX DNA Multi-Sample Ultra 2.0 Kit (Thermo Fisher Scientific Baltics UAB, Vilnius, Lithuania) on the KingFisher ™ Flex Purification System (Life Technologies Holdings Pte. Ltd., Singapore), following the manufacturer’s guidelines. DNA quantity was assessed by Qubit 4 fluorometer (Thermo Fisher Scientific, Waltham, MA, USA).

We used the Genome Variation Server to identify three polymorphisms in the CC16 (*SCGB1 A1*) gene: rs4963506, rs12270961, and rs3741240 [47]. Allele frequencies for rs4963506, rs12270961, and rs3741240 were examined to fulfill the Hardy–Weinberg Equilibrium.

CC16 genotyping was conducted in 96-well plates on QuantStudio ™ 12 K Flex Real-Time PCR System (Thermo Fisher Scientific, Waltham, MA, USA) using the pre-designed TaqMan® (Thermo Fisher Scientific, Waltham, MA, USA) allelic discrimination assays C__32321483_10 (rs4963506), C__31053318_10 (rs12270961), and C__25473445_10 (rs3741240).

Each reaction was carried out in 10 μl of a total reaction volume containing 5 µl of TaqMan Genotyping Master Mix, 0.5 μl of 20 × TaqMan assay, and 4.5 μl (approximately 20 ng) of genomic DNA. PCR conditions for rs4963506, rs12270961, and rs3741240 were the following: 60 °C for 30 s (pre-read stage), 95 °C for 10 min, 40 cycles of 95 °C for 15 s and 60 °C for 1 min (PCR stage), and a final post-read stage at 60 °C for 30 s. Non-template controls were included in triplicates for each reaction. In 15% of randomly selected samples, genotyping was replicated with a 100% accordance in results. QuantStudio 12 K Flex Software v1.5 (Thermo Fisher Scientific, Waltham, MA, USA) was used for genotype calling. In the presence of at least one variant allele genotype discrimination plots were automatically generated. All results were validated based on the multicomponent plots for each sample.

### Measurement of serum CC16 concentration

Serum samples for CC16 measurement were obtain when available on the first, and the fourteenth postnatal day. The serum samples were centrifuged at 3,000 rpm for 10 min and then the supernatants were stored at −80 °C. Upon the completion of the sample collection, the CC16 was measured in duplicate by a sandwich enzyme-linked immunosorbent assay kit, with a sensitivity of 0.046 ng/ml (Biovendor, Candler, NC, USA), as previously reported [22].

### Statistical analysis

Continuous variables were expressed as mean ± standard deviation or median (interquartile range), as appropriate. Comparisons in CC16 polymorphism genotypes and haplotypes were performed between neonates with RDS versus neonates without RDS, and between neonates with BPD versus neonates without BPD. Also, CC16 serum levels were examined according to CC16 polymorphisms. Comparisons of continuous variables were performed utilizing the student’s unpaired t-test or the non-parametric Mann–Whitney test. Categorical variables were expressed as n (percentage %) and compared with the chi-square test or Fisher’s exact test. Univariate associations of CC16 polymorphisms with RDS and BPD were evaluated, and odds ratios (OR) and 95% confidence intervals (CI) were calculated. A multivariate logistic regression analysis was used to examine the association of rs4963506, rs12270961, and rs3741240 CC16 polymorphisms with RDS and BPD, adjusted for gestational age, mode of delivery, and complete course of antenatal administration of steroids. The Bonferroni correction for multiple testing was utilized. Collinearity was examined with correlation matrix, and variables with significant collinearity, such as gestational age and birth weight, was examined separately in the model.

A power analysis was performed based on the reported incidences of CC16 polymorphisms in healthy adults [10]. With an estimated incidence of homozygous GG polymorphisms rs4963506, rs12270961, and rs3741240 of 43%, 63%, and 63% in healthy subjects, respectively, a sample of 100 neonates with RDS would adequetly detect 20–30% difference in the incidence of homozygous GG polymorphisms between neonates with and without RDS. All tests were two-sided and a p-value less than 0.05 was considered statistically significant (alpha 0.05). The data were analyzed using SPSS Statistics (IBM SPSS Statistics for Windows, Version 26.0. Armonk, NY, USA). LDlink was used to create the Linkage Disequilibrium plot (https://ldlink.nih.gov/?tab=ldmatrix), and SRplot to create the Linkage Disequilibrium heatmap (https://www.bioinformatics.com. cn/plot_basic_ LDheatmap_ plot_094_en).

## Results

During the study period, 187 neonates with a gestational age of 31.6 ± 2.6 weeks and a birth weight of 1760 ± 596 g were enrolled, of whom 125 (67%) developed RDS, and 16 (9%) developed BPD. All neonates survived to discharge. Neonates with RDS were of gestational age of 31.0 ± 2.7 weeks and birth weight of 1614 ± 614 g, as compared to 33.1 ± 1.6 weeks of gestational age and 2054 ± 433 g of controls (*p* < 0.001), respectively (Table 1). Among the neonates with RDS, 82 (66%) received surfactant, 68 (54%) required mechanical ventilation, and 16 (13%) developed BPD (Table 1).

**Table 1:** Neonatal characteristics in the total cohort and between neonates with and without RDS

	Overall (*n* = 187)	RDS (*n* = 125)	Non-RDS (*n* = 62)	*p*-value
Gestational age, weeks	31.6 ± 2.6	31.0 ± 2.7	33.1 ± 1.6	< 0.001
Birth weight, g	1760 ± 596	1614 ± 614	2054 ± 433	< 0.001
Small for gestational age	27 (15)	18 (14)	9 (15)	1.000
Sex. male	116 (62)	78 (62)	38 (61)	1.000
Mode of delivery, cesarean section	178 (95)	123 (98%)	55 (89)	0.007
Chorioamnionitis	4 (2)	3 (2)	1 (1)	1.000
Pregnancy-induced hypertension	22 (12)	15 (12)	7 (11)	1.000
Maternal gestational diabetes	32 (17)	22 (17)	10 (10)	0.840
Multiparity	87 (47)	59 (47)	28 (45)	0.881
Conception, *iv-vitro* fertilization	94 (50)	67 (54)	27 (44)	0.216
Antenatal steroids, complete	94 (50)	55 (44)	39 (63)	0.101
Prolonged rupture of membranes	33 (18)	25 (20)	8 (13)	0.309
Apgar 1 st minute	8 (8–8)	8 (7–8)	9 (8–9)	0.245
Apgar 5 th minute	9 (9–9)	9 (9–9)	10 (9–10)	0.064
Respiratory distress syndrome	125 (67%)	125 (100)	−	n/a
Ventilation	68 (36)	68 (54)	−	< 0.001
Surfactant	82 (44)	82 (66)	−	< 0.001
Mechanical ventilation, days	0 (0–1)	0 (0–1)	−	< 0.001
Non-invasive ventilation, days	1 (0–3)	2 (1–6)	−	< 0.001
Patent ductus arteriosus	25 (13)	18 (14)	7 (11)	0.652
Late-onset sepsis	27 (14)	16 (13)	11 (18)	0.383
Retinopathy of prematurity	8 (4)	5 (4)	3 (5)	1.000
Bronchopulmonary dysplasia	16 (9)	16 (13)	−	0.002
Length of stay	34 (17–50)	32 (17–49)	35 (19–56)	0.298

Continuous variables are expressed as mean ± standard deviation, or median (interquartile range). *P*-value of student’s t-test or Mann-Witney’s test. Categorical variables are expressed as n (percentage %). *P*-value of chi-square test or Fisher’s exact test

*RDS*; respiratory distress syndrome

### CC16 polymorphisms

**Fig. 1:**
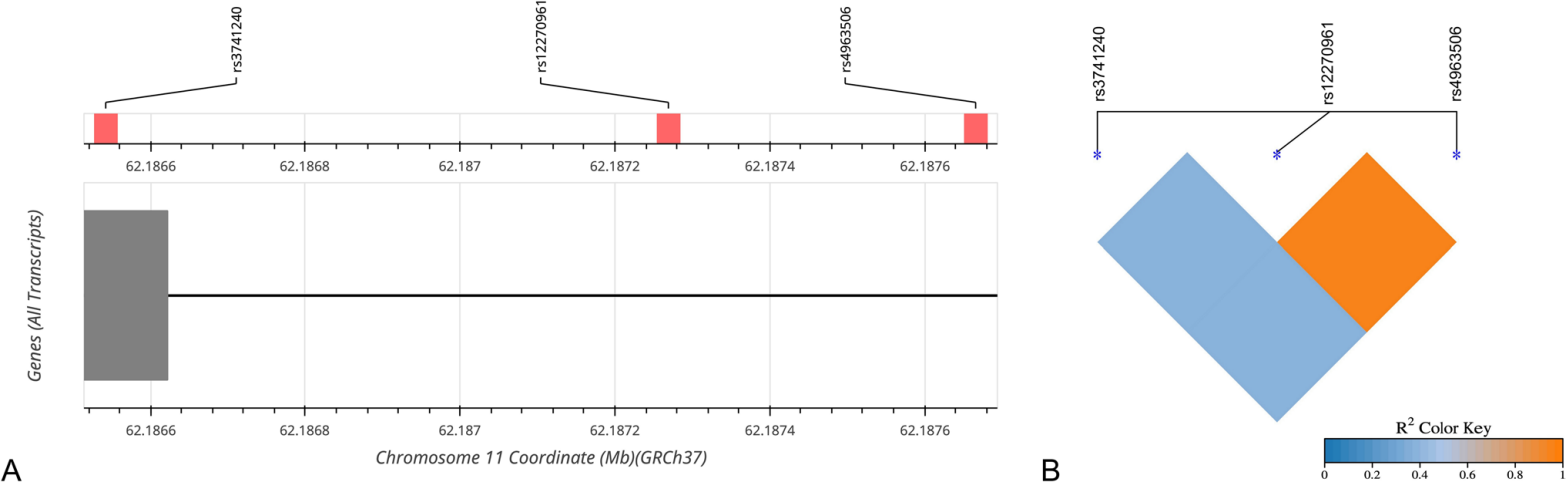
**A** Diagram of the CC16 (*SCGB1 A1*) gene located on chromosome 11q12.3. The rs4963506 is located in position chr11:62,420,194 (GRCh37), the rs12270961 in position chr11:62,419,797 (GRCh37), and the rs3741240 in position chr11:62,419,070 (GRCh37). **B** Linkage disequilibrium (R2) plot for CC16 polymorphisms. rs12270961 and rs4963506 are in nearly complete linkage disequilibrium. CC16, club cell secretory protein

The location and linkage of CC16 polymorphisms are shown in Fig. 1. All three CC16 polymorphisms, rs4963506, rs12270961, and rs3741240, were in Hardy–Weinberg Equilibrium. The polymorphisms rs4963506 and rs12270961 were in nearly complete linkage disequilibrium (Fig. 1). Genotyping of rs4963506, rs12270961 and rs3741240 was successful for 172, 168 and 174 samples, respectively. In neonates with RDS, the frequency of genotypes GG, GA, and AA for the rs4963506 polymorphism were 72%, 24%, and 4%, respectively, compared to 58%, 42%, and 0, respectively, in neonates without RDS (p = 0.018). The homozygous GG rs4963506 variant was associated with an increased risk of RDS (OR 1.26, 95%CI 1.02–1.75, *p* = 0.048) (Table 2A).

**Table 2:** Genotype frequencies of rs4963506, rs12270961, and rs3741240 polymorphisms between (A) neonates with and without RDS, and (B) between neonates with and without BPD

A	Overall	RDS	Non-RDS	OR (95%CI)	*p*-value
rs4963506	*n* = 172	*n* = 110	*n* = 62		
Genotype frequency					
* GG*	115 (67)	79 (72)	36 (58)		0.018
* GA*	52 (30)	26 (24)	26 (42)		
* AA*	5 (3)	5 (4)	−		
Homozygous GG versus GA/AA variant					
GG	115 (67)	79 (72)	36 (58)	1.26 (1.02–1.75)	0.048
GA/AA	57 (33)	31 (28)	26 (42)		
Allele	*n* = 344	*n* = 220	*n* = 124		
* G*	282 (82)	184 (84)	98 (79)	1.12 (0.89–1.41)	0.308
* A*	62 (18)	36 (16)	26 (21)		
rs12270961	*n* = 168	*n* = 108	*n* = 60		
Genotype frequency					
* GG*	108 (65)	76 (70)	32 (53)		0.013
* GA*	56 (33)	28 (26)	28 (47)		
* AA*	4 (2)	4 (4)	-		
Homozygous GG versus GA/AA variant					
GG	108 (65)	76 (70)	32 (53)	1.30 (1.01–1.70)	0.044
GA/AA	60 (35)	32 (30)	28 (47)		
Allele	*n* = 336	*n* = 216	*n* = 120		
* G*	271 (81)	179 (83)	92 (77)	1.15 (0.91–1.44)	0.199
* A*	65 (19)	37 (17)	28 (23)		
rs3741240	*n* = 174	*n* = 112	*n* = 62		
Genotype frequency					
* GG*	73 (42)	47 (42)	26 (42)		0.751
* GA*	89 (51)	56 (50)	33 (53)		
* AA*	12 (7)	9 (8)	3 (5)		
Homozygous GG versus GA/AA variant					
GG	73 (42)	47 (42)	26 (42)	1.00 (0.80–1.25)	0.997
GA/AA	101 (58)	65 (58)	36 (58)		
Allele	*n* = 348	*n* = 224	*n* = 124		
* G*	235 (68)	150 (67)	85 (69)	0.97 (0.82–1.14)	0.812
* A*	113 (32)	74 (33)	39 (31)		
B	Overall	BPD	Non-BPD	OR (95%CI)	*p*-value
rs4963506	*n* = 172	*n* = 14	*n* = 158		
Genotype frequency					
* GG*	115 (67)	9 (64)	106 (67)		0.847
* GA*	52 (30)	5 (36)	47 (30)		
* AA*	5 (3)	-	5 (3)		
Homozygous GG versus GA/AA variant					
GG	115 (67)	9 (64)	106 (67)	0.89 (0.31–2.54)	0.832
GA/AA	57 (33)	5 (36)	52 (30)		
Allele	*n* = 344	*n* = 28	*n* = 316		
* G*	282 (82)	23 (82)	259 (82)	1.01 (0.40–2.55)	0.981
* A*	62 (18)	5 (18)	57 (18)		
rs12270961	*n* = 168	*n* = 12	*n* = 156		
Genotype frequency					
* GG*	108 (65)	6 (50)	102 (65)		0.542
* GA*	56 (33)	6 (50)	50 (32)		
* AA*	4 (2)	−	4 (3)		
Homozygous GG versus GA/AA variant					
GG	108 (65)	6 (50)	102 (65)	0.64 (0.22–1.82)	0.547
GA/AA	60 (35)	6 (50)	54 (35)		
Allele	*n* = 336	*n* = 24	*n* = 312		
* G*	271 (81)	18 (75)	253 (81)	0.79 (0.33–1.89)	0.606
* A*	65 (19)	6 (25)	59 (19)		
rs3741240	*n* = 174	*n* = 14	*n* = 160		
Genotype frequency					
* GG*	73 (42)	3 (21)	70 (44)		0.133
* GA*	89 (51)	11 (79)	78 (49)		
* AA*	12 (7)	−	12 (7)		
Homozygous GG versus GA/AA variant					
GG	73 (42)	3 (21)	70 (44)	0.37 (0.10–1.30)	0.157
GA/AA	101 (58)	11 (79)	90 (56)		
Allele	*n* = 348	*n* = 28	*n* = 320		
* G*	235 (68)	17 (61)	218 (68)	0.72 (0.34–1.53)	0.409
* A*	113 (32)	11 (39)	102 (32)		

Categorical variables are expressed as n (percentage %). P-value of chi-square test or Fisher’s exact test

*RDS*; respiratory distress syndrome, *BPD*; bronchopulmonary dysplasia, *HWE;* Hardy–Weinberg Equilibrium, *OR*; odds ratio, *CI*; confidence intervals

Also, the frequency of genotypes GG, GA, and AA for the rs12270961 polymorphism were 70%, 26%, and 4%, respectively, in neonates with RDS, compared to 53%, 47%, and 0%, respectively, in neonates without RDS (*p* = 0.013). The homozygous rs12270961 GG variant was also associated with an increased risk of RDS (OR 1.30, 95%CI 1.01–1.70, *p* = 0.044). The genotype frequencies for rs3741240 polymorphisms did not vary by RDS status (Table 2A). Regarding the development of BPD, the genotype frequencies for all three polymorphisms (rs4963506, rs12270961, and rs3741240) did not vary by BPD status (Table 2B).

The haplotypes of rs4963506, rs12270961, and rs3741240 polymorphisms are shown in Table 3. No differences in haplotypes between neonates with and without RDS, or with and without BPD were detected.

**Table 3:** The haplotypes of rs4963506, rs12270961, and rs3741240 polymorphisms between (A) neonates with and without RDS, and (B) between neonates with and without BPD

A rs4963506/rs12270961/rs3741240	RDS (*n* = 107)	Non-RDS (*n* = 61)	*p*-value
G/G/G	97 (91)	58 (95)	0.709
G/G/A	6 (5)	3 (5)	
A/A/G	1 (1)	−	
A/A/A	3 (3)	−	
B rs4963506/rs12270961/rs3741240	BPD (*n* = 12)	Non-BPD (*n* = 156)	*p*-value
G/G/G	12 (100)	143 (91)	1.000
G/G/A	−	9 (6)	
A/A/G	−	1 (1)	
A/A/A	−	3 (2)	

Categorical variables are expressed as n (percentage %). P-value of chi-square test or Fisher’s exact test

*RDS*; respiratory distress syndrome, *BPD*; bronchopulmonary dysplasia

In regression analysis, a significant association with an increased risk of RDS was detected for the homozygous GG rs4963506 variant (OR 3.03, 95%CI 1.36–6.75, *p* = 0.021), and the homozygous GG rs12270961 variant (OR 2.88, 95%CI 1.31–6.28, *p* = 0.024), but not for the homozygous GG rs3741240 variant, after adjusting for gestational age, mode of delivery, and complete course of antenatal steroid administration (Table 4). No associations between BPD and the genotype frequencies of any of the three polymorphisms (rs4963506, rs12270961, or rs3741240) were detected.

**Table 4:** Regression analysis of the association of homozygous GG rs4963506, rs12270961, or rs3741240 variant with RDS, adjusted for gestational age, birth weight, mode of delivery, and antenatal administration of steroids

	OR	95%CI	*p*-value
Homozygous GG rs4963506 genotype	3.03	1.36–6.75	0.021
Gestational age	1.45	1.15–1.83	0.006
Mode of delivery	4.37	0.70–27.08	0.339
Complete course of antenatal steroids	4.44	0.79–11.06	0.303
Homozygous GG rs12270961 genotype	2.88	1.31–6.28	0.024
Gestational age	1.42	1.13–1.79	0.006
Mode of delivery	4.14	0.66–25.95	0.387
Complete course of antenatal steroids	4.22	0.70–10.45	0.306
Homozygous GG rs3741240 genotype	1.54	0.73–3.24	0.762
Gestational age	1.39	1.11–1.73	0.012
Mode of delivery	4.50	0.74–27.19	0.303
Complete course of antenatal steroids	3.85	0.61–9.22	0.306

RDS, respiratory distress syndrome; OR, odds ratio; CI, confidence intervals

### CC16 polymorphisms and serum CC16

In the 42 neonates with available serum CC16 measurements, the homozygous GG rs4963506 variant was associated with higher CC16 levels on the first postnatal day (11.5 ± 4.3 ng/mL) compared to the GA/AA rs4963506 variants (9.3 ± 1.8 ng/mL, *p* = 0.046). No differences were detected in neonatal serum CC16 levels, on the first or the fourteenth postnatal day, between the genotypes of either rs12270961 or rs3741240 polymorphisms (Table 5).

**Table 5:** Serum CC16 levels on the first and the fourteenth postnatal day on neonates with compared to without RDS, according to the genotypes of the rs4963506, rs12270961, and rs3741240 polymorphisms

	First day		Fourteenth day	
	CC16 levels (ng/ml)	*p*-value	CC16 levels (ng/ml)	*p*-value
RDS	15.42 ± 8.41	0.007	5.44 ± 2.10	0.859
Non-RDS	9.31 ± 3.50		5.32 ± 1.80	
rs4963506 genotype				
GG	11.5 ± 4.3	0.046	5.6 ± 1.8	0.134
GA/AA	9.3 ± 1.8		4.5 ± 2.0	
rs12270961 genotype				
GG	11.3 ± 4.3	0.431	5.4 ± 1.7	0.762
GA/AA	10.3 ± 3.0		5.2 ± 2.4	
rs3741240 genotype				
GG	12.5 ± 4.7	0.122	5.7 ± 1.7	0.503
GA/AA	10.2 ± 3.9		5.3 ± 2.2	

*RDS*; respiratory distress syndrome, *CC16*; club cell secretory protein

## Discussion

The current study investigated the genotype frequencies for rs4963506, rs12270961, and rs3741240 CC16 polymorphisms and CC16 serum concentration in a cohort of preterm neonates and explored their association with RDS and BPD. Our findings suggest that the homozygous GG rs4963506 and rs12270961 variants were significantly associated with an increased risk of RDS, whereas the homozygous GG rs4963506 variant was also associated with higher serum levels of CC16 compared to the GA/AA rs4963506 variants, on the first day of age. No association was found between any of the three examined CC16 polymorphisms and BPD, in our cohort.

Previous studies investigating the genotype frequencies for CC16 polymorphisms in children and adults were mainly focused on their association with asthma, asthma control, and atopia, with inconsistent findings [14, 23, 24]. While several studies found a positive correlation between the incidence of asthma and the rs3741240 polymorphism [13, 25, 26], other studies found no such correlation [10, 15, 27]. One study estimated that children carrying the risk genotype AA for rs3741240 had nearly four times greater odds of asthma than those with the GG genotype [14]. A recent meta-analysis including data from 19 case–control studies reported that those individuals with the AA or AG genotype for CC16 rs3741240 had 1.3 times greater odds of asthma compared to the GG genotype (pooled OR 1.29, 95%CI 1.08–1.54) [23]. Finally, in a most recent study in adults, CC16 rs3741240 polymorphism was not associated with asthma, asthma subtypes, asthma control, exacerbations, or respiratory symptoms [10]. However, uncontrolled asthma was found to be associated with the rs12270961 CC16 G-allele [10].

Gene studies investigating the genotype frequencies for CC16 polymorphisms in neonates are lacking; therefore, comparisons to the present study are limited. Our findings suggest a strong association between the homozygous GG rs4963506 and rs12270961 variants with an increased risk of RDS. We also found that neonates with the homozygous GG rs4963506 variant had higher serum levels of CC16 compared to those with the GA/AA rs4963506 variants, on the first day of age. Evidence from previous studies suggests that CC16 is detectable in cord blood, showing a postnatal surge and reaching its highest level on the first day of life [5]. Gestational age has been associated with circulating levels of CC16 in neonates, with lower levels detected in preterm neonates [5, 6, 28]. Prolonged rupture of membranes was associated with lower cord blood CC16 levels [29], while reduced CC16 concentrations in the tracheobronchial aspirates were reported in extremely premature neonates with a fetal inflammatory response syndrome [30], suggesting a link between systemic inflammation and pulmonary downregulation of CC16 in early postnatal life [31]. Moreover, postnatal lung injury leads to an elevation of the production of CC16 in the alveoli [28], and a subsequent increase of circulating CC16 concentrations due to the leakage across the bronchoalveolar-blood barrier [5, 7]. Evidence has shown that both RDS and invasive mechanical ventilation contribute significantly to an increase in CC16 concentration in preterm infants within the first postnatal days [7, 22, 28]. Finally, lower cord blood CC16 concentrations have been significantly associated with the development of BPD [5, 6, 29]. Persistently low circulating CC16 levels have been associated with the development of BPD [5], although evidence is inconsistent as higher levels of circulating CC16 have been also measured in infants subsequently developing BPD [7]. Significantly lower levels of CC16 were also detected in the bronchoalveolar lavage fluid from ventilated neonates with BPD, compared to non-BPD neonates, indicating a positive correlation between bronchoalveolar lavage fluid CC16 levels at birth and BPD [3].

The strength of our study is that it provides novel insights into the genetic lineage of CC16 polymorphisms in preterm neonates and the development of serious respiratory diseases, namely RDS and BPD. However, further studies are required to validate whether the identification of rs4963506, rs12270961, and rs3741240 CC16 polymorphisms could potentially help detect neonates at risk for increased respiratory morbidities. The limitations of our study should be acknowledged. First, our cohort consisted mainly of very preterm neonates, with only a small proportion of them born below 28 weeks of gestational age. These extremely preterm neonates suffer at most from respiratory morbidities and would likely benefit the most from the detection of high-risk CC16 polymorphisms. Also, the serum levels of CC16 were not available for the entire neonatal cohort, therefore, caution is needed in the interpretation of the association of CC16 polymorphisms and the circulating levels of CC16. Finally, as per the study design that included both ventilated and non-ventilated neonates, we were unable to measure CC16 concentrations in the bronchoalveolar lavage fluid that would help to better understand the kinetics of CC16 in relation to CC16 polymorphisms.

## Conclusion

In preterm neonates, the homozygous GG rs4963506 and rs12270961 variants were both substantially associated with a higher risk of RDS, whereas no correlation was observed among CC16 polymorphisms and BPD. On the first day of life, the homozygous GG rs4963506 variant was also linked to higher serum levels of CC16 than the GA/AA rs4963506 variants. Further studies are warranted to validate our findings and explore the potential role of CC16 polymorphism detection in neonates at risk of respiratory morbidities.

## Supplementary information

Below is the link to the electronic supplementary material.

ESM 1 (DOCX 33.3 KB)

## Data Availability

No datasets were generated or analysed during the current study.

## Abbreviations

BPD: Bronchopulmonary dysplasia
CI: Confidence interval
CC16: Club cell secretory protein
RDS: Respiratory distress syndrome
OR: Odds ratio
